# Introduction to the Special Issue on Wars and Disasters: Advancing Care during Times of Crisis

**DOI:** 10.3390/ebj5030026

**Published:** 2024-09-02

**Authors:** Leopoldo C. Cancio

**Affiliations:** U.S. Army Institute of Surgical Research, Fort Sam Houston, TX 78234, USA; leopoldo.c.cancio.civ@health.mil

**Keywords:** burns, inhalation injury, disaster medicine, military personnel, armed conflict

## Abstract

Civilian mass-casualty disasters and armed conflict share many features, including the fact that both maximally challenge multidisciplinary burn teams. Rigorous training is required to build teams and systems that can respond effectively. One of the critical but potentially overlooked components of readiness for crisis care is a robust clinical research program. Rather than stalling progress, disasters and conflict over the last 100 years consistently energized advances in care. This was made possible by the hard work of our predecessors *to learn from the crisis in the midst of the crisis*, and resulted in significant reductions in postburn mortality. Now, further work is needed not only to maintain these improvements in mortality, but also to understand the long-term functional outcomes and to improve the quality of life of burn survivors. Clinical research programs to address these issues must be established now, so that we are optimally prepared for the next conflict or disaster.

## 1. Introduction

Any review of the substantial progress in the care of burn patients over the past century must take into account the role of both armed conflict and civilian mass-casualty disasters in energizing these improvements. Certainly, the concept that military experience serves as a training ground for surgeons has long been recognized. In his 1904 lecture on the history of medicine and surgery, Sir Thomas Allbutt (inventor of the clinical thermometer) remarked “I would remind you again how large and various was the experience of the battlefield, and how fertile the blood of warriors in rearing good surgeons” [1].

The same is true of the effect of wartime experience on other members of the multidisciplinary burn team. During the 20th century, technological advances meant that the battlefield now included, for the first time, large numbers of casualties with burns and inhalation injuries. We saw, for example, the use of chemical agents during WWI, mechanized Blitzkrieg warfare during WWII, and nuclear weapons at Hiroshima and Nagasaki. These changes challenged those who would take care of combat casualties with burns, while also contributing to civilian mass-casualty preparedness. Several examples of military–civilian synergy in burn care come to mind (Table 1).

## 2. Underhill

One example of military–civilian synergy in burn care is the work of Yale pharmacologist and toxicologist Frank Underhill. In 1930, he published experience with the casualties from the Rialto Theater fire in New Haven, CT of 1921. He described the process of hemoconcentration, or “anhydremia”, seen in burn patients. He correctly attributed this finding to the loss of a substance from the blood, similar to plasma across the microvasculature, arguing against the prevailing theory that early death after burn was caused by release of injurious toxins from the damaged tissue. He proposed the administration of sodium chloride solutions intravenously, and the serial measurement of hemoglobin levels as a guide to resuscitation [4].

Underhill’s thinking about burn shock actually began earlier, during WWI—not because of burns, but because of experience with chemical warfare agent casualties. He had observed that inhalation of chlorine, phosgene, and chlorpicrin caused massive pulmonary edema, which “induces a marked concentration of the blood…in both abnormal states…(E)ither an irritant gas or heat leads to an extreme inflammatory reaction and destruction of tissue” [3].

## 3. Churchill and Lyons

A second example of the interplay between military and civilian mass-casualty burn care is the Cocoanut Grove fire of November 1942 [8]. By that time, the U.S. had already been at war with the Axis powers for almost a year. The importance of burns during this new war was signaled by UK experience during the Battle of Britain of July-October 1940—the campaign which resulted in the establishment of a burns unit by Sir Archibald McIndoe at the Queen Victoria Hospital in East Grinstead, UK, as well as the famous Guinea Pig Club for burn survivors [5,6]. The Japanese attack on Pearl Harbor of December 1941 generated 1178 wounded in action, of whom “about sixty percent” had burns [14]. This led the U.S. National Research Council in January 1942 to set up a Subcommittee on Burns (led by Allen Whipple, best known for pancreatic surgery) and to initiate burn research programs at several U.S. hospitals. At the time of the Cocoanut Grove fire, two such programs were already under way at the Massachusetts General Hospital (MGH), one on wound infections and one on burn physiology [7].

Because of those nascent programs and the well-established academic mindset of the MGH staff, the hospital was well prepared not only for the clinical care of those casualties, but also for analysis of and publication on that care. The result was a monograph which, in my view, represents the first comprehensive report on the multidisciplinary team approach to burn care, encompassing most of the problems encountered by burn patients, from airway management to rehabilitation [9]. Among the authors of the Cocoanut Grove monograph were Edward Churchill, who wrote the foreword, and Champ Lyons, who wrote the paper on the use of antimicrobials. Soon after the fire, these authors were deployed to North Africa where they rapidly translated their findings to combat casualty care on the battlefield, likely saving countless lives.

Churchill was a thoracic surgeon, and the chair of the West Surgical Service (and later the General Surgery Service) at the MGH. He is well known for performing, along with MGH colleague and Cocoanut Grove co-author Oliver Cope, the first mediastinal parathyroidectomy on Captain Charles Martell. As described in his book *Surgeon to Soldiers*, although he did not directly care for Cocoanut Grove casualties, he recognized the importance of the event and went to the bedside to learn about the care. He wrote the following:

*“As soon as I recognized the magnitude of the Cocoanut Grove Disaster, I called Allen [Whipple], who was in New York. This was about 1:30 in the morning. I told him the situation, that the M.G.H. and the Boston City Hospital were full of casualties. He got out of bed and caught a five o’clock train…he was there before the last dressings were applied. He also alerted observers from the Office of the Surgeon General” [10]*.

Soon thereafter Churchill was deployed as a Colonel in the U.S. Army Medical Corps, assigned as the Consultant to the Surgeon General for the Mediterranean Theater. His focus in that theater was on setting up blood banks for transfusion of whole blood, aeromedical evacuation, the use of penicillin, and eliminating tannic acid from the treatment of burn wounds [15].

For penicillin, Churchill turned to Lyons [16]. Now a Major in the U.S. Army Medical Corps, Lyons was principally responsible for introducing penicillin into the North African battlefield [17]. His subsequent efforts in the U.S. included running a unit in Utah for treatment of combat casualties with infected wounds, and then a Wound Study Unit at Halloran General Hospital, Staten Island, NY for the same purpose [18]. After the war, this unit moved to Fort Sam Houston, TX in 1947 as the U.S. Army Surgical Research Unit (USASRU; later, the U.S. Army Institute of Surgical Research, USAISR) to continue its work on infected wounds and antibiotics, but at that time had yet to become a burn unit.

## 4. Mason, Moncrief, and Pruitt

A third example of civilian–military collaboration is how the fledging USASRU became the second burn unit in the U.S. In August 1949, the Soviet Union detonated its first atomic weapon. U.S. planners were aware that the use of such weapons at Hiroshima and Nagasaki had generated tens of thousands of burn survivors, a situation for which the U.S., or any country, was ill prepared. The USASRU was therefore directed to focus on clinical burn research. Careful documentation of the cause of death in burn patients [11] and integrated laboratory–clinical investigations on the microbiology of infected burn wounds [12] led, in 1964, to arguably the single most important development, from a mortality perspective, in burn history—an effective topical antimicrobial, mafenide acetate, for the prevention of invasive bacterial burn wound infection [13]. Since Zora Janžekovic’s seminal publication in 1970 [19], the importance of topical wound care has yielded, in part, to that of early surgical excision and grafting. But the contribution by Basil Pruitt, Arthur Mason, John Moncrief, and colleagues on mafenide acetate is one of numerous advances which stemmed from a need to prepare for armed conflict, and which also positively impacted civilian care around the world.

## 5. Discussion

In reflecting on the above examples, several themes come to mind. Underhill represents the ability to make connections between seemingly different clinical phenomena—in his case, between pulmonary edema caused by WWI war gases and burn shock caused by skin burns. Churchill, Lyons, and the other Cocoanut Grove authors represent the importance of clinical research programs and of the rapid deployment of advances in times of crisis. Pruitt and colleagues represent the value of integrated laboratory and clinical research, directed at solving the problems of real patients.

Where do we go from here? From a civilian perspective, large-scale fire disasters are relatively uncommon [20], but I would argue that this merely heightens the risk of difficulty should a disaster occur and increases the need for more training. From a military perspective, we know that burns will be a feature of combat casualty care on the future battlefield, comprising about 5–10% of such casualties [21]. Furthermore, we are concerned that future large-scale combat operations (LSCO) against a peer or near-peer adversary will generate many more thermally injured combat casualties than recent conflicts. Evacuation of these casualties off the battlefield will likely be delayed, creating a requirement for prolonged field care of burn patients and other casualties [22].

The ability of any one nation to greatly expand burn capacity in times of crisis is limited; for example, during the early months of the recent war in Iraq (Operation Iraqi Freedom) in 2003, a daily U.S. burn bed census revealed that the mean number of open burn ICU beds in the 70 participating U.S. burn centers was 167 [range, 83–239] [23]. This means that during LSCO, non-burn centers, such as civilian trauma centers, will have to absorb some of the burn-care burden. During peacetime, our patients have benefitted from the establishment of burn centers, the regionalization of burn care, and the development of subspecialty burn expertise. But such specialization generates a need to develop training programs for our non-burn colleagues, so they can assist during a crisis.

From a research perspective, we also must recognize that the extraordinary improvement in postburn survival contributed by our predecessors has plateaued in recent years. The problem of postburn mortality has been supplanted by the problem of survivorship. Thus, how best to document and to improve long-term outcomes in burn survivors has become an imperative clinical research objective [21]. Along with the need to focus on long-term outcomes is a need to expand the clinical research role to other members of the burn team. In this Special Issue, we highlight the diversity of the burn team by including medical intensivists, an infectious diseases physician, a physician assistant, dieticians, nurses, an occupational therapist, a pharmacist, and a psychologist among the authors.

In conclusion, the groundbreaking authors cited above demonstrated the importance of multidisciplinary clinical research programs to advancing the care of the injured, particularly during times of mass-casualty disaster or armed conflict. This Special Issue provides a selection of recent advances and current problems in burn-care applicable to both scenarios.

## Figures and Tables

**Table 1:** Relationship between military and disaster medicine in burn care.

Event or Problem	Date	Impact on Burn Care	References
Facial trauma, WWI	1914-8	Development of facial reconstructive surgery by Gillies.	[2]
Chemical warfare, WWI	1915-8	Pathophysiology of inhalation injury; influenced Underhill’s understanding of burn shock.	[3]
Rialto Theater fire	1921	Pathophysiology of burn shock; i.v. fluid resuscitation.	[4]
Battle of Britain, WWII	1940	Establishment of burn unit by Gillies’ student, McIndoe; reconstructive techniques for burns; survivor peer support. Blood for Britain program; plasma for resuscitation.	[5,6]
Pearl Harbor, WWII	1941	Founding of NRC Subcommittee on Burns and of burn research programs in U.S. hospitals, to include MGH and BCH.	[7]
Cocoanut Grove fire	1942	At MGH: evaluation of i.v. plasma for shock; lung injury; infections [use of penicillin]; others.	[8,9]
North African campaign, WWII	1943	Introduction of penicillin to the battlefield.	[10]
Cold War; USSR detonates atomic weapons	1949	Establishment of first burn units in U.S.; epidemiology of burn mortality; pathophysiology of invasive burn wound infection; topical antimicrobials; others.	[11,12,13]

NRC, U.S. National Research Council. MGH, Massachusetts General Hospital. BCH, Boston City Hospital.
